# Validation of computed tomography angiography using mean arterial pressure gradient as a reference in stented superior mesenteric artery

**DOI:** 10.1007/s00261-020-02700-6

**Published:** 2020-08-09

**Authors:** Niklas Lundin, Leena Lehti, Olle Ekberg, Stefan Acosta

**Affiliations:** 1 Division of Medical Radiology, Department of Translational Medicine, Lund University, Malmö, Sweden; 2 Department of Clinical Sciences, Lund University, Malmö, Sweden; 3 Vascular Center, Department of Cardio-Thoracic and Vascular Surgery, Skåne University Hospital, S205 02 Malmö, Sweden

**Keywords:** Computed tomography angiography, Mean arterial pressure gradient, Superior mesenteric artery, Stent, Mesenteric atherosclerotic disease

## Abstract

**Purpose:**

The aim of this prospective study was to validate the diagnostic performance of computed tomography angiography (CTA) in endoprosthesis stenosis in the superior mesenteric artery (SMA) using mean arterial pressure (MAP) gradients during angiography as a reference method.

**Methods:**

Twenty-nine patients with mesenteric atherosclerotic disease underwent 45 paired measurements of endoprosthesis stenosis in the SMA with CTA and MAP gradients between March 2009 and July 2015. The grade of endoprosthesis stenosis in the SMA at CTA using the TeraRecon Aquarius workstation was correlated with MAP gradients.

**Results:**

Grade of endoprosthesis stenosis in the SMA (*r* = 0.37, *p *= 0.013) correlated with MAP gradients. The intraclass correlations between the first and second CTA rater was 0.76 (95% CI 0.56–0.87) for estimation of grade of endoprosthesis stenosis in the SMA. The area under the receiver operating characteristics curve was 0.79 for diagnosis of significant endoprosthesis stenosis in the SMA at CTA for different threshold values using MAP gradient of ≥ 10 mmHg as reference. Sensitivity, specificity and positive predictive value for endoprosthesis stenosis in the SMA ≥ 50% at CTA were 52.4% (95% CI 31.0–73.7), 87.5% (95% CI 74.3–100.0) and 78.6 (95% CI 57.1–1.00), respectively.

**Conclusion:**

Grading endoprosthesis stenosis in the SMA with CTA performed fair when using trans-stenotic MAP gradient as reference. Software development towards reduction of endoprosthesis artefacts may result in more accurate CTA assessment of the narrowest part.

## Introduction

Endovascular therapy for mesenteric atherosclerotic disease has become an established, minimally invasive method. However, restenosis within the endoprosthesis develops often with reported re-intervention rate as high as 33% [1], and mortality after acute stent occlusion in the superior mesenteric artery (SMA) has been reported to be 50% [2].

Angiography with measurement of trans-stenotic mean arterial pressure (MAP) gradient during angiography is considered to be the most accurate method for diagnosis of a significant SMA stenosis [1, 3]. Since angiography is invasive and exposes patients and personnel to radiation, the European Society of Vascular Surgery (ESVS) recommend colour Doppler ultrasound (CDU) of the mesenteric arteries as first line examination [4] and performance of CDU when using MAP gradient as a reference was recently found to be good [5]. However, CDU is a more operator-dependant modality in comparison to CTA [6]. Ultrasound is challenged by the rapid evolution of high-resolution computed tomography angiography (CTA) imaging available around the clock, especially in the subacute and acute setting. The diagnostic performance of CTA for evaluation of endoprosthesis stenosis in the SMA using a modern software tool is unknown.

The main aim of the present study was to evaluate the diagnostic performance of CTA with TeraRecon Aquarius workstation using trans-stenotic MAP gradient as reference in patients with endoprosthesis in the SMA for mesenteric atherosclerotic disease.

## Patients and methods

### Study population

Prospective evaluation of trans-stenotic MAP gradients and CTAs in patients with acute and chronic mesenteric atherosclerotic disease and endoprosthesis stenosis during the same in-hospital stay was routinely performed according to the department’s memorandum between March 1st, 2009 and July 31st, 2015. Ethical approval was therefore waived by the regional ethical review board. The protocol consisted of colour doppler ultrasound and CTA after endovascular SMA stenting and clinical visits at 3 months, 1 year, and yearly thereafter. Asymptomatic patients with peak systolic velocity > 3.3 m/s on colour doppler ultrasound or SMA stenosis ≥ 50% underwent angiography with MAP gradient measurements, and subsequently re-stented if MAP gradient ≥ 10 mm Hg. Eighty-five SMA stenting procedures were performed. Among 65 trans-stenotic MAP gradient measurements, 45 CTAs for simultaneous evaluation of in stent stenosis were performed. Estimation of renal function by measurement of serum creatinine and calculation of glomerular filtration rate (GFR) was always done prior to intervention. The endovascular revascularization technique has previously been described [1].

### Mean arterial pressure gradient measurement

Results after stenting were controlled by completion angiography as well as measurement of the MAP gradient across the stented arterial segment. Measurement of MAP gradient was performed using the 6-Fr introducer with its tip in the abdominal aorta near the SMA and the 4 Fr Cobra slip catheter coaxially placed through the introducer, with the tip distally to the stented arterial segment. The arterial pressures of the aorta and the SMA were recorded simultaneously using an electronic recorder (Siemens SC 9000 XL). The zero level was set prior to examination for each patient. The absolute values of systolic, diastolic and mean arterial pressure, in the aorta and SMA were recorded, respectively. The gradient was the difference between the aortic and SMA (distal to endoprosthesis) mean arterial pressure and when ≥ 10 mmHg it was classified as a hemodynamically significant stenosis [1].

### Computed tomography angiography and radiation dose

The CT scanners used were from four different vendors. A dual-phase protocol consisting of an unenhanced phase and early arterial contrast enhanced phase was used.

At all CTA examinations volume computed tomography dose index (CTDI_vol_) and dose-length product (DLP) were registered by the CT scanner. The effective dose (ED) for the study was calculated from DLP multiplied by the mean of the ED/DLP conversion factor for the abdomen (0.15 mSv/mGy × cm) and pelvis (0.13 mSv/mGy × cm), i.e. 0.14 mSv/mGy × cm based on the International Commission on Radiological Protection (ICRP) 103 tissue weighting factors [7]. The radiation variables are shown in Table 1.

**Table 1:** Radiation variables in computed tomography arterial phase scans

Variable	Contrast scan
CTDI_vol_ (mGy)	6.4 (2.8-11.0)
Dose-length product (mGy*cm)	332 (120-577)
Effective dose (mSv)	4.6 (1.7-8.1)

Median values (2.5 and 97.5 percentiles) are given. CTDI_vol_ = volume computed tomographic dose index. The Effective (mSv, milli Sievert) dose is the sum of non-contrast and arterial phase contrast scan radiation doses in the CT study evaluating the total radiation effect to organs or tissues, weighted for their different sensitivity to radiation

### Assessment of the stenosis at CTA

**Fig. 1:**
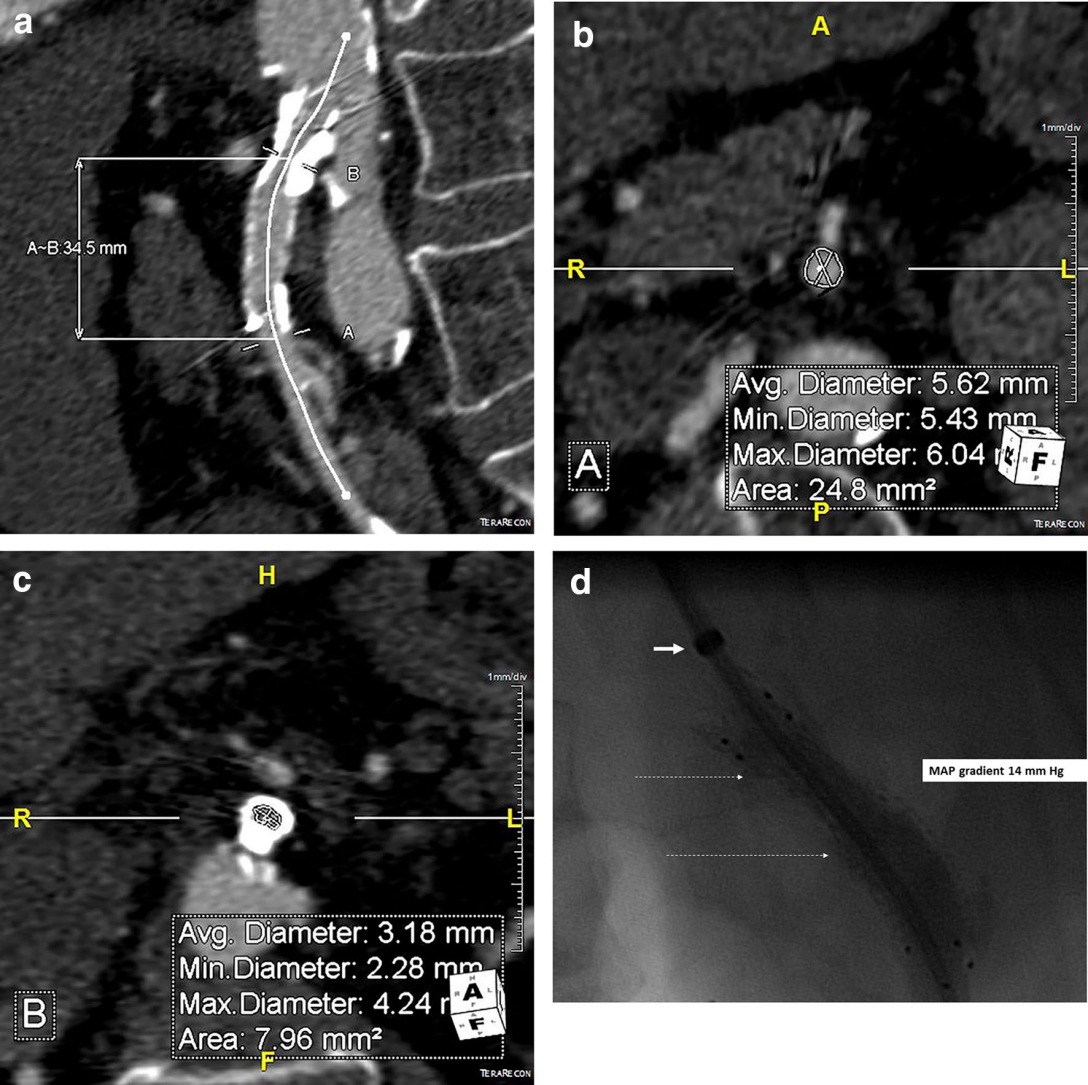
69-year-asymptomatic female patient with stented SMA. Follow-up colour doppler ultrasound showed a peak systolic velocity of 6.5 m/s. TeraRecon 3D CTA image indicates the centerline of flow (Fig. 1a). Measurements of arterial diameters were always performed in the plane perpendicular to its course. The respective average diameters were measured at the largest diameter distal to the occlusive lesion and the endoprosthesis (Fig. 1b) and narrowest intraluminal part of the stented arterial segment (Fig. 1c). SMA stent stenosis was calculated to be 43%. Angiography (Fig. 1d) was performed under general anaesthesia. The SMA was accessed percutaneously from the right brachial artery. The tip of the introducer (thick short arrow) ends at the origin of the SMA from the aorta. The hard atherosclerotic stenotic lesion at the backwall of the SMA (between dashed arrows) has caused a recoil of the endoprosthesis after previous stentgrafting (best visualized in this un-subtracted image), resulting in a MAP gradient of 14 mm Hg. After subsequent re-balloon dilatation, the MAP gradient was reduced to 5 mm Hg. Since then, the patient has remained symptom-and re-intervention-free during the seven-year follow-up

Assessment of SMA endoprosthesis stenosis at CTA (Fig. 1a–c) were performed blinded to MAP gradients (Fig. 1d), and independently by two radiologists, a resident and a senior interventional radiologist, by using the TeraRecon Aquarius software (TeraRecon Inc, North Carolina, USA) indicating the centerline of flow (Fig. 1a) at a radiological workstation. In all CTA studies the SMA was examined in three projections (axial, coronal and sagittal) and the smallest luminal diameter as well as the largest diameter distal to the restenosis in the stented SMA was estimated. Measurement of arterial diameters were always performed in the plane perpendicular to its course (Fig. 1a–c). The grade of endoprosthesis stenosis was calculated according to North American Symptomatic Carotid Endarterectomy Trial (NASCET) [8] method [the difference between largest diameter distal to the occlusive lesion and the endoprosthesis (Fig. 1b)] and narrowest intraluminal part of the stented arterial segment (Fig. 1c) divided with the same largest diameter (Fig. 1b) converted to percent]. A ≥ 50% stenosis was considered as a significant endoprosthesis stenosis in the SMA. For testing intra-rater reliability ten CTA studies were randomly chosen and re-assessed three months later by each radiologist.

### Statistical Methods

Data management and statistical analysis were performed using SPSS for Windows, version 25.0 (SPSS Inc. Chicago, IL). Differences in proportions were analysed using Chi-square test. Continuous variables were expressed in medians and interquartile ranges (IQR) or range. Correlations were expressed with Pearson or Spearman Correlation Coefficient. Inter- and intra-rater reliability of CTA variables were expressed as intraclass correlation (ICC) with 95% confidence intervals (CI), and a value of > 0.7 was regarded as satisfactory [9]. Diagnostic performance of different threshold values for SMA endoprosthesis stenosis using trans-stenotic MAP gradient as reference (≥10 mm Hg) resulted in a receiver operating characteristics (ROC) curve and expressed with area under the curve (AUC) value. The AUC values were interpreted as follows: 0.90–1.0 = excellent; 0.80–0.90 = good; 0.70–0.80 = fair; 0.60–0.70 = poor; 0.50–0.60 = failure. Sensitivity, specificity, positive predictive value, negative predictive value and accuracy were calculated with 95% CI for endoprosthesis stenosis in the SMA ≥ 50% at CTA after cross tabulation against MAP gradient ≥10 mm Hg. A p value < 0.05 was considered significant.

## Results

### Patient characteristics

This study included 45 paired examinations with MAP gradient and CTA in 29 patients who were stented in the SMA due to acute or chronic mesenteric ischemia. Median age at time of intervention was 72 years (IQR 68–79). Nineteen (65%) patients were women and ten (35%) were men. Among the 29 patients who received the index revascularization procedure, 12 had SMA occlusion and 17 had high-grade stenosis. The corresponding nature of lesions in the coeliac trunk were 12 and 16, respectively. One patient had no significant stenosis in the coeliac trunk. Adjunctive bowel resection to SMA stenting was performed in 6.7% (3/45) in those examined with CTA, compared to 15.0% (6/40) in those not undergoing CTA evaluation (*p *= 0.21).

### Endovascular therapy

There were 29 (64.4%) endovascular re-interventions, 26 (76.5%) out of 34 interventions in women versus 3 (27.3%) out of 11 in men (*p *= 0.003). The indications for re-interventions were asymptomatic stent restenosis (*n *= 18; 62%) and symptomatic (*n *= 11; 38%) stent restenosis (*n *= 9)/occlusion (*n *= 2). Twenty-six (57.8%) interventions were performed due to clinical symptoms of mesenteric ischemia and three (6.7%) needed a bowel resection. The endovascular procedures were performed under general and local anaesthesia in 18 (39.1%) and 27 (60.0%) interventions, respectively. The main atherosclerotic occlusive lesion was treated with a balloon expandable stent in 24 interventions, using a 6 mm (*n *= 2), 7 mm (*n *= 10) or 8 mm (*n *= 12) diameter stent, versus a stent graft in 21 interventions, using a 6 mm (*n *= 16), 7 mm (*n *= 3) or 8 mm (*n *= 2) diameter stent graft (p<0.001). Sixteen (72.7%) stent grafts versus thirteen (54.2%) balloon expandable stents were used in re-interventions (*p *= 0.19).

### Inter- and intra-rater reliability of CTA variable measurements

The ICCs between the first and second rater were 0.80 (95% CI 0.64–0.89), 0.97 (95% CI 0.94–0.98) and 0.76 (95% CI 0.56–0.87) for estimation of the smallest luminal diameter at the stenosis, largest luminal diameter distal to the SMA stenosis and grade of SMA stenosis, respectively, in the 45 CTAs.

The ICCs for the first rater after repeat assessment were 0.98 (95% CI 0.91–0.99), 0.96 (95% CI 0.86–0.99) and 0.94 (95% CI 0.74–0.98), respectively. The ICCs for the second reader were 0.98 (95% CI 0.94–1.00), 0.98 (95% CI 0.93–1.00) and 0.96 (95% CI 0.84–0.99), respectively.

### Correlation of CTA variables and MAP gradients

**Fig. 2:**
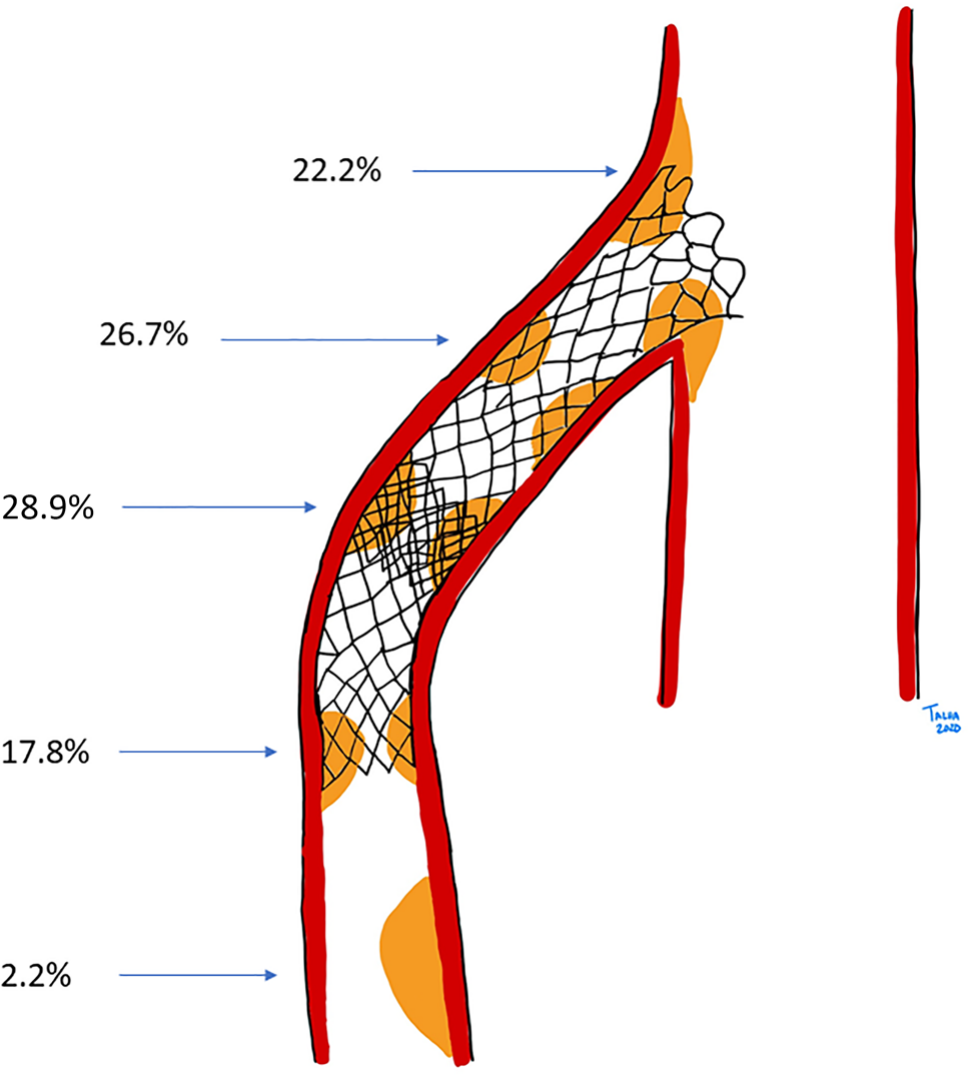
Schematic drawing on location of restenosis in the endoprosthesis or native SMA. *Artist: Talha Butt*

The narrowest luminal part of the SMA according to CTA was located at the proximal edge of the endoprosthesis (near the SMA origin from the aorta, *n *= 10; 22.2%), middle part of the endoprosthesis (*n *= 12; 26.7%), overlapping parts of endoprostheses (*n *= 13; 28.9%), distal edge of endoprosthesis (*n *= 8; 17.8%) and distal to endoprosthesis in native SMA (*n *= 1; 2.2%) (Fig. 2). One (2.2%) endoprosthesis or native SMA displayed no identified localized stenosis. The median grade of endoprosthesis or native SMA stenosis was 43% (IQR 33–55), and significant grade of endoprosthesis or native SMA stenosis (≥ 50%) was found in 14 (31.1%) cases. The grade of SMA endoprosthesis or native SMA stenosis was correlated with MAP gradients (*r* = 0.37; *p *=  0.013). The smallest luminal diameter of the endoprosthesis or native SMA was inversely correlated to MAP gradients (*r* = − 0.37; *p *= 0.013).

### Diagnostic performance of significant endoprosthesis or native SMA stenosis at CTA

**Fig. 3:**
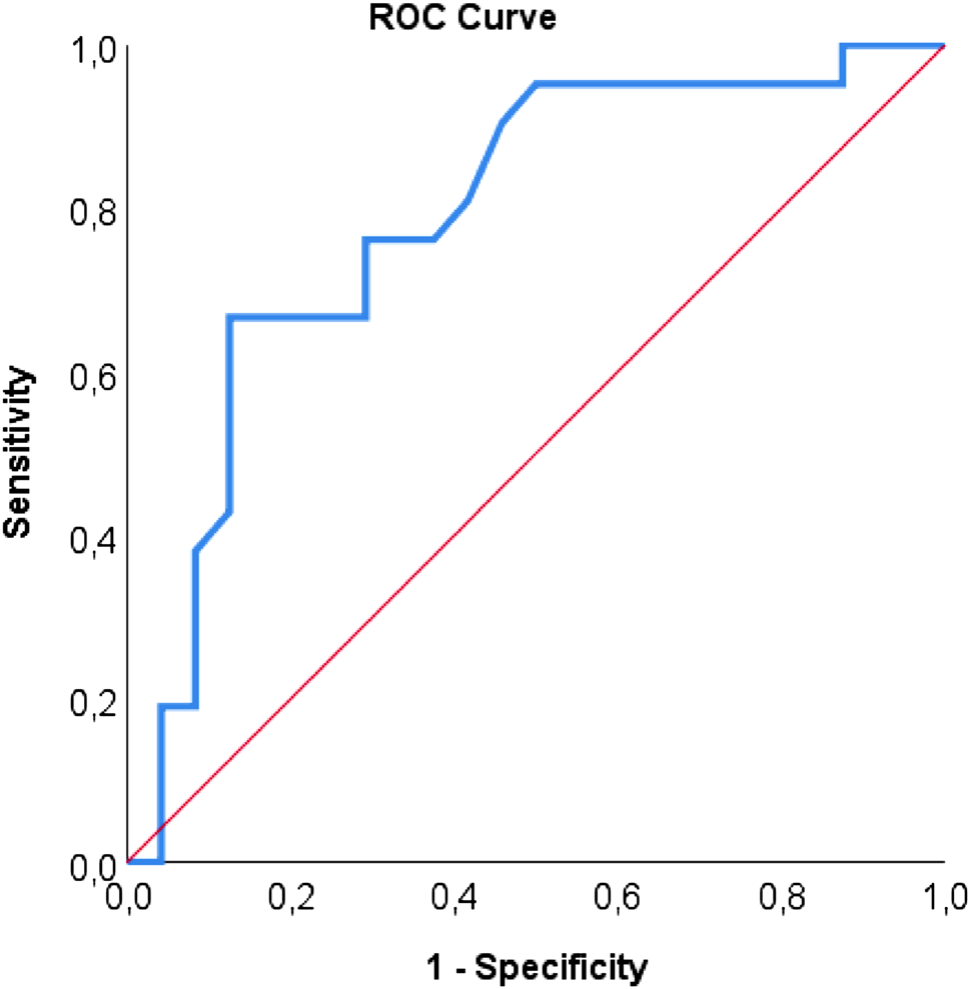
Diagnosis of significant endoprosthesis stenosis in the SMA for different threshold values at CTA using MAP gradient of 10 mm Hg as reference is illustrated in a ROC curve. The AUC was 0.79

Significant endoprosthesis or native SMA stenosis (≥ 50%) at CTA correlated with significant MAP gradients (≥10 mm Hg) (*r* = 0.43; *p *= 0.003). Diagnosis of significant endoprosthesis or native SMA stenosis for different threshold values of SMA stenosis using MAP gradient of 10 mm Hg as reference resulted in a ROC curve with an AUC value of 0.79 (Fig. 3). Sensitivity, specificity, positive predictive value, negative predictive value and accuracy for endoprosthesis or native stenosis in the SMA ≥ 50% at CTA were 52.4% (95% CI 31.0–73.7), 87.5% (95% CI 74.3–100.0), 78.6 (95% CI 57.1–1.00), 67.7 (95% CI 51.3–84.2) and 71.1 (95% CI 57.9–84.4), respectively.

## Discussion

The present prospective study showed that grade of SMA endoprosthesis stenosis at CTA was correlated with angiographic MAP gradients. The performance characteristics of CTA can be summarized as fair, and similar to CDU performance [5]. To pursue and report pressure measurements before and after deployment of endoprosthesis is very important in the management of patients with suspected chronic mesenteric ischemia as MAP gradients reflect clinical severity of SMA stenosis [10]. A successful mesenteric revascularization should not only be accompanied by reduction of MAP gradient towards zero, but also to immediate symptom relief [11].

In one report, CTA resulted in better diagnostic accuracy for significant SMA stenosis over magnetic resonance angiography and ultrasound, when compared to angiography [12]. In comparison with ultrasound and magnetic resonance angiography, CTA can be performed around the clock for fast image generation without the need of expert technicians [13]. In the present study, the use of TeraRecon Aquarius software at radiological workstation with centerline of flow measurements [14] was found to be another technological advantage when assessing grade of SMA stenosis. The potential drawbacks of CTA are the iodine contrast exposure and the risk of contrast-induced renal insufficiency, contrast allergy, and the exposure to radiation. However, CT angiography followed by endovascular intervention for acute SMA occlusion, resulted in double dose of iodine contrast compared to CT angiography alone, but was not found to increase the risk of iodine contrast-induced renal failure [15].

The overall fair diagnostic performance for CTA for grading SMA endoprosthesis stenoses in relation to MAP gradients can be improved. First of all, four different CT scanners with non-identical performance characteristics and x-ray source settings were used in the study, resulting in a great variation of radiation parameters in arterial phase scans as outlined in Table 1. There are a number of artefacts to be aware of and to reduce, related to metallic implants [16] at CT. Depending on the size and composition (atomic number) of the endoprosthesis, different degrees of X-ray attenuation and physical effects will occur. Endoprosthesis used for treatment of occlusive SMA lesions are usually made of nitinol alloy (nickel titanium) or stainless steel (mainly chromium content except for iron), which have a relatively low atomic number that may cause relatively minor beam hardening in comparison with other metallic hardware such as platinum or tantalum [17]. Highly attenuating materials such as metal stents and calcifications inherently cause beam hardening, and when small structures are evaluated with CTA, corresponding to the smallest intraluminal diameter in the present study, partial volume averaging can also be problematic [18]. Both beam hardening and the partial volume averaging effect contribute to the stent-associated blooming artefacts, resulting in a thicker appearance of the stent struts and subsequent underestimation of the narrowest intraluminal diameter, resulting in increased percentage of false determination of significant endoprosthesis stenosis at CTA [19].

A first step for reduction of artefacts appears to be to use projection-based metal artefact reduction (MAR) algorithms, a software application that can be used retrospectively. The MAR algorithms preserves the iodine contrast enhancement, in addition to reducing bright and dark band artefacts from metallic hardware. Several MAR algorithms can be developed [20] and tested specifically for metal artefact reduction of SMA endoprostheses. Of note, all CT images in the present study were assessed without a MAR algorithm, since the raw data from the CT scans were not saved for future software reconstructions. An alternative is to beforehand decide to use dual-energy CT technique, characterized by data acquisition at two different high-energy spectra, for reduction of the effects of beam hardening. This technique may be associated with simultaneous reduction of iodine contrast enhancement, decreasing the diagnostic performance of the assessment of vascular patency. Since the occlusive atherosclerotic lesions in the SMA often are heavily calcified, there are concerns of low specificity for significant arterial stenoses at dual-energy CT, which has been found in patients with symptomatic peripheral arterial disease [21]. The increased radiation exposure for the patient compared to standard CT is also a drawback of this technique.

In this prospective study, 76% underwent MAP gradient measurements and 53% of all endovascular SMA stent procedures were evaluated with both MAP gradient measurements and CTA. The reason for not adhering to the protocol may be multiple, such as emergency procedures in a non-hybrid angio suite not suitable for endovascular procedures, unawareness of the study protocol among physicians not writing a referral letter to the radiologists for CTA, and short in-hospital stay after SMA stenting with patient transferred back to the referral hospital. In addition, patients not examined with CTA postoperative underwent bowel resection in a higher percentage. In view of the 6-year-long study period, adherence to the study protocol was acceptable. Determination of inter-rater reliability among CTA readers was also a scientific strength to increase generalizability of the results.

In conclusion, CTA performed fair in evaluation of significant SMA endoprosthesis stenosis when using trans-stenotic MAP gradient as reference. There is room for improvement for more accurate CTA assessment of SMA endoprosthesis stenosis.

## Data Availability

SPSS database.
